# Efficacy of ultrasound guided dry needling as an adjunct to conventional physical therapy for patients with jumper’s knee: A randomized controlled trial

**DOI:** 10.3389/fsurg.2022.1023902

**Published:** 2022-11-04

**Authors:** Faiza Sharif, Ashfaq Ahmad, Syed Amir Gilani, Raham Bacha, Asif Hanif, Muhammad Asim Arif

**Affiliations:** ^1^ University Institute of Physical Therapy, The University of Lahore, Lahore, Pakistan; ^2^ Faculty of Allied Health Sciences, The University of Lahore, Lahore, Pakistan; ^3^ University Institute of Radiological Sciences and Medical Imaging Technology, University of Lahore, Lahore, Pakistan; ^4^ University Institute of Public Health, Faculty of Allied Health Sciences, University of Lahore, Lahore, Pakistan

**Keywords:** jumper's knee, patellar tendinopathy, ultrasonogaphy, dry needling, clinical trial

## Abstract

**Background:**

Jumper’s knee is a degenerative condition among athletes, and it has been treated with conventional physical therapy (CPT). Ultrasound guided dry needling (USG-DN) is a relatively new technique to explore clinical outcomes in patients with tendinopathy.

**Methods:**

This parallel group, single-blinded randomized controlled trial was carried out involving 94 athletes with clinically diagnosed jumper’s knee, divided into an intervention group (USG-DN + CPT, *n *= 47) and a control group (CPT, *n *= 47). Participants received a 4-week programme; the intervention group received ultrasound guided patellar tendon dry needling (DN) in conjunction with CPT. The control group received only CPT. The visual analog scale (VAS), Victorian institute of sports assessment-Patellar tendinopathy (VISA-P) questionnaire, Lysholm scale, Knee injury and osteoarthritis outcome score (KOOS) and ultrasonographic features of patellar tendinopathy were evaluated at baseline,1 week, 2 weeks, and 4 weeks. The data were analyzed through SPSS-26.

**Results:**

The study found statistically significant differences (*P *< 0.05) regarding VAS, Lysholm, VISA-P, and KOOS scales at baseline, 1st, 2nd, and 4th week post-intervention. Within-group differences also showed statistically significant results after the intervention. There were significant results observed in ultrasonographic outcomes between both groups at 1 month post-intervention (all *P *< 0.05).

**Conclusion:**

The results of the current study suggest, ultrasound guided DN of patellar tendon in combination with CPT reduced pain, improved function, and showed a tendency to decrease tendon thickness in patients with patellar tendinopathy.

**Clinical Trial Registration Number:**

(IRCT20210409050913N1). Dated: 17.04.2021. https://www.irct.ir/user/trial/55607/view.

## Background

Patellar tendinopathy (PT), also known as jumper’s knee, is a clinical syndrome of anterior knee pain localized to the inferior pole of the patella associated with loss of function due to mechanical loading (1, 2). There is great variation in the prevalence of PT with the overall prevalence in nonelite players from different sports being 8.5% (3), which increases in elite sports (4). This condition is two times higher in male nonelite athletes than in female athletes (3, 4). According to the available scientific evidence (5), it is unclear whether changes in tendon tissues have a strong association with pain, but it shows that these changes are correlated with functional loss of tendons, enhancing the probability of developing a symptomatic picture when changes are significant (6). With a high frequency, duration, and intensity of quadriceps contraction in activities requiring repetitive knee flexion and extension, the patellar tendon can develop micro-tears at its insertion on the inferior patellar pole.

The symptoms of patellar tendinopathy are treated with conservative treatments with therapeutic exercises being the most effective (7). The initial choice for patellar tendinopathy treatment includes cessation of the aggravating activity until symptoms resolve, stretching and strengthening exercises of the quadriceps, hamstrings, and heat therapy and nonsteroidal anti-inflammatory drugs (NSAIDs) and patellar strap to minimize stress on the patellar tendon. The results after conservative treatment are not promising and symptoms frequently recur (8, 9). Short-term tendon pain can be controlled with isometric contractions in the early stages of tendon rehabilitation (10). High-demand exercises such as eccentric contractions and heavy slow resistance are used to achieve collagen remodeling and long-term tendon function improvement (7). Surgery can be an alternative in severe cases that failed to respond to conservative treatments. However, due to high risks and complications, the results of surgery are unreliable (11–13).

Few minimally invasive techniques have been suggested for health care professionals that may be helpful for tendinopathy due to their advantages and less risk, such as dry needling (DN) (1, 14) and electrotherapeutic invasive modalities such as percutaneous needle electrolysis (PNE) (15–18). Currently, research has focused on regenerative therapies due to the quick improvement of symptoms and a probable regeneration of the injured tendon (1, 16, 19).

Ultrasound guided DN and autologous blood injection showed promising results in patients with patellar tendinosis (1). In an animal experiment, a study reported that ultrasound guided DN of the rat supraspinatus tendon causes a transient healing response followed by a return to or improvement in normal tendon properties, indicating that DN can be a potential treatment option in the human tendon as well (20). Tendon DN involves repeated fenestration of the injured tendon, which is assumed to disintegrate the chronic degenerative process and can trigger localized bleeding and fibroblastic proliferation. This in turn leads to the ordered collagen synthesis and ultimately healing of the tendon. However, both optimal dosage and retention time of the needle are yet to be determined (21). So, we hypothesized that ultrasound guided DN in junction with conventional physical therapy (CPT) would provide superior results/outcomes than isolated CPT. The findings of a case study conducted in 2021 showed that there is improvement in pain and function after quadricipital tendon dry needly in jumper’s knee patients (22). Similarly, a systematic review conducted by Girorgi et al. in 2022 concluded that there is level B evidence to support the use of tendon DN in combination with eccentric exercises (EE) to improve pain and function in tendinopathies (23). Literature showed a lack of high-quality controlled trials and few studies had methodologies deficiencies, so the present study aims to determine the effects of ultrasound guided DN on pain, functional disability, and ultrasonographic changes in patients with jumper’s knee and determine which is the most effective for patients with jumper’s knee.

## Materials and methods

### Ethical approval

The prospective, parallel randomized controlled trial was approved by The Institutional Review Board committee of the University of Lahore (IRB-UOL-FAHS/829-I/2021). The trial is registered at the Iranian registry of clinical trials (IRCT20210409050913N1 Registered on April 17, 2021). The first participant was recruited in the current trial on April 17, 2021. All the participants involved in the trial duly signed the written informed consent before their participation. Ethical considerations were taken into account, and prior to treatment, the therapist provided the participants with a form stating that minor bruising, pain or slight bleeding is expected in small number of participants. The trial was conducted at the Physiotherapy department, University teaching hospital, The University of Lahore Pakistan.

The study was guided by the Consolidated Standards of Reporting Trials (CONSORT) statement and included the CONSORT checklist.

### Participants and study design

From April 2021 to July 2022, participants with jumper’s knee or patellar tendinopathy referred by an orthopedic surgeon at the University Teaching Hospital, University of Lahore were asked to participate in the trial. In this study we diagnosed jumper’s knee as tender patellar tendon, palpation tenderness of the superior insertion of the patellar tendon, and score of <80 on the Victorian Institute of Sports Assessment for PT (VISA-P) questionnaire and ultrasonographic examination of the patellar tendon.

Patients were included if they met the following criteria: Participants of either gender between the age of 18–45 years with a confirmed diagnosis of jumper’s knee for more than 1 month and pain intensity of ≥3.0 on the visual analog scale (VAS) (0–10) while ascending and descending stairs and high pain intensity in single leg decline squat test.

Participants were excluded if they had surgery around the knee joint within the last 6 months, chronic knee joint disease, knee pain management with corticosteroid injections in the last 3 months, relative and absolute contraindications for needling such as allergies, hypersensitivities, presence of implants, knee joint inflammation, presence of calcific deposits in the proximal patellar tendon, with a known history of fractures around the knee joint, any bleeding disorders and opioid use in last 48 h or any other intervention taken for jumper’s knee.

### Interventions

Interventions consisted of two treatment sessions per week and a follow-up period of 1 month (total of eight sessions). Each session lasted 45 min and was executed by the same therapist with 11 years of experience in musculoskeletal physical therapy.

#### Ultrasound guided dry needling (experimental group)

In this group, patients were treated with ultrasound guided DN and CPT that included an exercise program, activity modification, and physiotherapy modalities i.e., hot pack, ultrasound, and transverse friction massage. The DN technique was performed by a certified DN physical therapist. The ultrasound was performed by a senior radiologist simultaneously with DN application by a physical therapist. The assessor was the same one who assessed the symptoms and function of the jumper’s knee. The ultrasound guided the procedure for accurate needle insertion within the patellar tendon, enhancing the treatment efficacy, and reducing the risk of accidental injury to other surrounding tissues. The treatment area of the knee and ultrasound probe was disinfected with an antiseptic solution (70% isopropyl alcohol) to prevent infections. The appropriate treatment area was selected based on the ultrasonographic examination of the tendon areas exhibiting degenerative changes.

All the participants were advised not to receive any other treatment for jumper’s knee. However, NSAIDs were permitted if patients had unbearable pain and there was no schedule of outcome assessment in the next 48 h. There was a complete record of documentation of all such concomitant treatments (such as name and dosages of the drugs and treatment duration) received by the patients during the study.

For ultrasound guided DN as well as pre-procedure and post-procedure assessment of the patellar tendon, high-frequency ultrasound equipment (Xario Premium, Toshiba, Japan) and a linear probe (7–14 MHz) were used. The assessments under ultrasound were performed in accordance with the Musculoskeletal Ultrasound Technical Guidelines (MUTG): Knee, defined by the European Society of Musculoskeletal Radiology (EuSMR) (24). The assessment on the ultrasound of the longitudinal section of the patellar tendon from the origin of its insertion, whereas the transverse section included the patellar pole, body, and insertion of the patellar tendon on the tibial tuberosity, by positioning the patient in supine lying or sitting with 20° of knee flexion and pillow was placed under the knee for patient’s comfort. The target area of the involved tendon was selected and assessed with the presence of degenerative signs in accordance with the medical diagnosis of jumper’s knee. These degenerative signs included tendon thickness and hypoechoic areas.

Specific 25-gauge (0.25 × 25 mm) stainless steel DN needles were applied during the intervention by considering the approach and thickness of the patellar tendon. During the procedure, the DN needle reached the appropriate involved areas with focal degenerative tendon changes. The three needles were inserted during the whole treatment session and the insertions lasted for 3 s each. The total number of needle insertions can be varied from 20 to 30 passes, depending on the area of tendon degeneration under ultrasound guidance. These needle insertions changed the chronic degenerative process into an acute condition that is more likely to heal (25). The rest of the treatment session had the same interventions as given to the conventional group.

#### Conventional physical therapy

This group received only CPT. Cycling on a stationary bicycle with minimum resistance for 5–10 min was performed as an active warm-up. Static stretching of hip flexors, quadriceps, hamstring, and calf muscles was performed for a period of 30 s at least 3–4 times per day (26, 27) before and after the exercise session. The patients performed pain-free partial weight-bearing eccentric squats on a 25° decline board for 3 sets of 15 repetitions twice daily and as the pain subsided, the decline board angle increased, and exercises changed from bilateral eccentric to unilateral eccentric and then to concentric–eccentric contractions. Squatting was performed at 60°–70° knee flexion (28–30). The straight leg raises, side lying hip abduction/hip adduction, and prone hip extension (around the world) were the strengthening exercises for proximal hip and thigh musculature, performed initially with no weight combined with the decline eccentric squats and then progressed to 2 s concentric leg lift, followed by 4 s eccentric leg lowering (31, 32). Speed was increased during the concentric–eccentric phase and finally to the ballistic type (jump squats). In the last week of rehabilitation, the pain subsided and to prepare the patients for return to sports activities, an initial 10% body weight of the patients was used for the strengthening exercises with a weighted vest and then 5 kg weight was increased during progression (30, 33). Jumping activities were then added to this routine which progressed from double leg to single leg. The rehabilitation program also included 10 min of moist heat pack, application of pulsed ultrasound and deep transverse friction massage (34) of the patellar tendon for 5–10 min (Table 1) (35–37). The participants were advised to wear knee straps and modify activities (38).

**Table 1:** Rehabilitation exercises for patellar tendinopathy.

Weeks	Rest	Eccentric exercises	Transverse friction massage	Stretching exercises (30 s × 3–4×)
1	No jumping or running; can ride a bike, do pool work	Around the world eccentric lowering leg raises (4 way) increase weight by 1# each week)	5–10 min firmly 1–2 × a day	Hip flexors, quadriceps, hamstrings, and heel cords before/after activity
	No sports-specific training	Eccentric squats on decline board 15 reps × 3 sets 1–2 × a day/		
2	Initiate jumping squats in short range	Around the world eccentric lowering leg raises (4 way) (increase weight by 1# each week)	5–10 min firmly 1–2 × a day	Continue stretching as above
	No sports-specific training	Progress to upright decline board squats		
3	Cycle, aquatic exercises	Upright squats on decline board double leg to single leg	As required	Continue stretching as above
4	Progressive return to jumping/squatting/jump boxes	Jumping squats with single leg	As required	Continue stretching as above
	Initiate sports-specific training with gradual return to sporting events	Upright squats on decline board with 50#		
		Jumping squats with one leg on with maximal resistance		

X, times per day; reps: repetition; #: number.

## Outcome measurement

A written questionnaire consisting of questions about baseline data and the pathology of jumper’s knee was prepared. Different questionnaires and assessment tools [VAS, VISA-P, Lysholm, and Knee injury and osteoarthritis outcome score (KOOS) scales] were given to each participant with ample time to complete the questionnaires. The participants were measured at baseline, 1 week, 2 weeks, and 4 weeks post-randomization. Pain and function were assessed using VAS, VISA-P questionnaire, Lysholm knee scoring, and KOOS scales, respectively. Furthermore, a sonographic assessment of the patellar tendons of all the participants was also performed.

## Primary outcome measure

### Knee pain intensity

The anterior knee pain was assessed using a VAS (VAS ranging from 0 to 10, where 0 indicates no pain and 10 means worst possible pain). Pain was assessed at baseline and then at the 1st, 2nd, and 4th week post-treatment.

## Secondary outcome measures

### Functional disability

The severity of jumper’s knee was measured with VISA-P questionnaire. This scale comprises eight questions, with a maximum score of 100, indicating that the person is asymptomatic and fully functional whereas fewer scores show symptoms of patellar tendinopathy and functional limitation (39, 40). This scale has been reported valid and reliable tool for patients with jumper’s knee (41). The 13 points on the VISA-P scale are used to consider the minimal clinically important difference in athletes with jumper’s knee (42).

Lysholm comprises eight items and is scored on a scale of 0–100 assessing knee-related symptoms. These scores integrate both objective and subjective data. Objective data is clinically assessed by the physician and subjective functional data is obtained from the patients (43). Points of 95–100 on the scale are considered excellent, 85–94 points as good, 65–84 points as fair, and poor for less than 65 points (44).

Functional disability was assessed using The KOOS. It is a knee-specific instrument, developed to assess the patients’ opinions about their knee-associated problems. The KOOS evaluates both short-term and long-term consequences of knee injuries. It holds 42 items in five separately scored subscales: pain, other symptoms, function in daily living (ADL), function in sport and recreation (Sport/Rec), and knee-related quality of life (QOL). It is an extension of the WOMAC Osteoarthritis Index. The five patient-relevant subscales of KOOS are scored separately: pain (nine items), symptoms (seven items), ADL function (17 items), sport and recreation function (five items), and quality of life (four items) (45).

### Sonographic assessment of the patellar tendon

The thickness of the tendon was measured in the longitudinal section. Therefore, to measure the entire length of the patellar tendon from the inferior margin of the patella to the tibial tuberosity, and then find its mid-portion by dividing the entire length by 2. The epitendon and paratenon were not included in the measurement. The echogenicity and fibrillar echo pattern of the patellar tendon structures were evaluated both in the longitudinal and the transversal scans in comparison to Hoffa’s fat pad.

### Sample size

Consecutive sampling was used to recruit participants for the trial. The sample size was calculated based on the data provided by the pilot study. The pilot study was performed on 20 patients and sample size calculation was based on the Lysholm score of this study using the mean Lysholm score in the experimental group (70.5 ± 20.695), and in the control group (55.50 ± 11.07) using 80% power of test, 95% confidence interval, and 5% margin of error. The 96 participants were randomized into two groups, with 48 patients in each group adding a 20% dropout rate. This sample size was also sufficient for the VISA-P score and KOOS scales.

### Randomization and blinding

After baseline evaluation, the eligible participants were randomly allocated to both groups using a computer-generated randomized method, created prior to data collection by an independent researcher. The randomization allocation was sealed in opaque envelopes. The physical therapist opened the envelope and proceeded according to the group allocation. The assessor and biostatistician were blinded to the participant’s treatment allocation until the completion of the study. Participants were blinded to group allocation and were advised to not reveal any treatment experience to the outcome assessment examiner. This cannot make sure complete blinding; a questionnaire was given to participants at the end of the study. The participants were asked through email “Do you know which treatment you got?” The possible responses were: “needling treatment,” “no needling treatment,” or “I don’t know.”

Participants were informed about the needling intervention with moderate pain, and that if there was unbearable pain during the treatment, they must let the researcher know to stop the treatment immediately. The physical therapist providing the treatments could not be blinded, however, he/she was asked not to reveal the allocation of the patients during the intervention or follow-up sessions. Validity, reliability, and translation of the Lysholm scale had also been performed for the current trial (46).

## Statistical analysis

Descriptive statistics of age, gender, weight, height, and body mass index (BMI) were included in the mean, and SD, and is summarized in Table 2. US outcomes included changes in tendon thickness (mm), the thickness of the central hypoechoic area (mm), the presence of a central hypoechoic area (yes/no), and color Doppler activity (0–4). This way of quantifying color Doppler activity and measuring tendon thickness has previously been assessed regarding reliability and agreement in lateral epicondylitis in the common extensor tendon of the elbow (47). The outcome variables were evaluated with the Shapiro–Wilk test for normal distribution before the statistical analysis. Between and within-group analyses of the two treatment groups were carried out by analysis of variance (ANOVA) mixed model for repeated measures with Bonferroni *post hoc* pairwise comparisons in case of normal distribution. All analyses were carried out “per intent-to-treat.” The statistical level of significance was evaluated at *P* < 0.05 using a two-tailed test. SPSS (Statistical Package for Social Science) version 26.0 was used for Statistical analysis at 95% confidence interval.

**Table 2:** Baseline demographic characteristics.

	USG-DN + CPT group (*n *= 47)	CPT group (*n *= 47)	Total *N* (94)	*P-*value
Age (years)a	20.50 ± 2.42	21.62 ± 1.82	21.06 ± 2.1	0.21
Weight (kg)	68.62 ± 4.95	68.37 ± 6.38	68.5 ± 5.7	0.12
Height (cm)	171.25 ± 11.24	160.75 ± 8.85	166 ± 10.0	0.14
BMI (kg/cm^2^)^a^	23.73 ± 3.81	26.76 ± 4.20	25.2 ± 4.0	0.44
Females^b^	15 (31.9)	18 (38.2)	33 (35.1)	0.32
Duration of symptoms, mo	31.5 ± 31.3	22.8 ± 15.9	26.9 ± 24.3	0.63
Ultrasound				
Tendon thickness, mm	7.5 ± 0.23	7.6 ± 0.13	7.3 ± 2.0	
Thickness of central hypoechoic area, mm	4.42 ± 0.28	4.62 ± 0.30	4.7 ± 2.7	
Central hypoechoic area present, *n* (%)^b^	45 (95.7%)	46 (97.8%)	91 (96%)	
Color Doppler activity (grade 0–4)	2.55 ± 1.23	0.63 ± 0.94	2.3 ± 1.5	

Values are expressed as the mean ± standard deviation or number of participants.

BMI, body mass index; USG-DN, ultrasound guided dry needling; CPT, conventional physical therapy.

^a^ Data are expressed as mean ± standard deviation unless otherwise indicated.

^b^ Data expressed as number (%).

## Results

**Figure 1:**
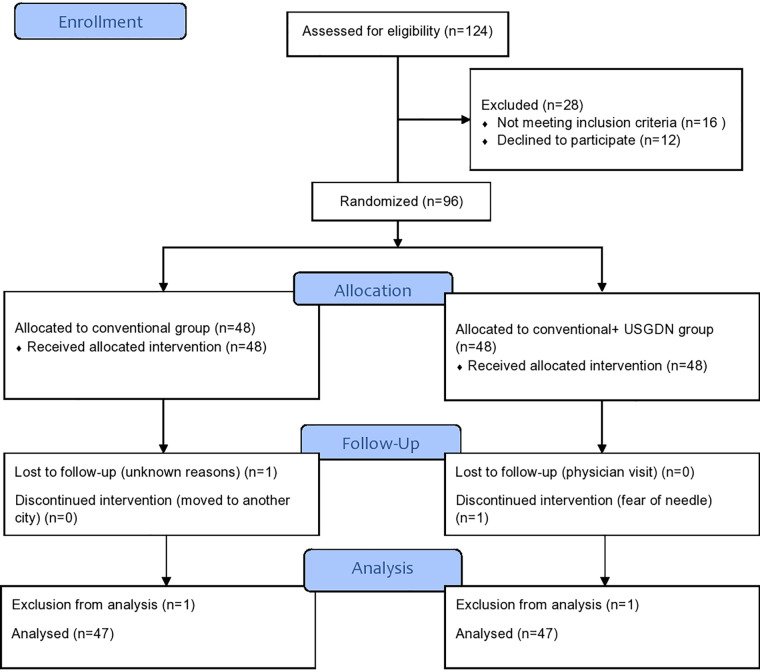
The overall summary of Consort diagram and study flow chart.

The screening of 124 patients with clinically diagnosed jumper’s knee was performed for possible participation in the study. After the exclusion of 28 participants, 96 fulfilled the inclusion criteria of the study. The 48 participants each were allocated to the USG-DN + CPT group and the CPT group. During the study, two participants dropped out and 94 participants completed the follow-up of 4 weeks. The whole data was normally distributed with the absence of any outliers, and baseline characteristics in each group were balanced and statistically insignificant for all variables. The overall schematic flow of the patient’s sample is given in CONSORT guidelines (Figure 1). There were no statistically significant differences between both groups in terms of the demographic and clinical characteristics at baseline.

## Outcome measures

Results showed a significant reduction in the VAS and signs of patellar tendinopathy and an improvement in VISA-P, Lysholm, and KOOS scales in both groups. There were statistically significant differences regarding VAS, VISA-P, Lysholm, and KOOS scales between the two groups at baseline and 2 and 4 weeks after intervention (*P*-value ≤ 0.05).

### Pain intensity

VAS in the USG-DN + CPT group was significantly lower than in the CPT group at all time points post-intervention Table 3. After the 1st week, pain decreased in the USG-DN + CPT group vs. the CPT group (1.2 ± 0.15 vs. 7.00 ± 0.90 points on VAS) which was higher after 2nd week (5.20 ± 0.75 vs. 7.20 ± 0.75 on VAS). After 4 weeks the pain intensity differed from baseline by 5.2 ± 0.68 in USG-DN + CPT group and 1.6 ± 0.15 in the CPT group.

**Table 3:** Outcome data for VAS, VISA-P, Lysholm and KOOS scales.

Outcome	Time (mean ± SD)				Intra group effect^a^	
	Baseline	First week	Second week	Fourth week	Time	Group-time
Group	Mean (SD)	Mean (SD)	Mean (SD)	Mean (SD)	*F* (DF); *P-*value (eta^2^)	*F* (DF); *P-*value (eta^2^)
VAS						
USG-DN group	8.20 ± 0.75	7.00 ± 0.90	5.20 ± 0.74	3.00 ± 1.43	*F* (6.38; 6.72) = 621.07	*F* (−1.740; 1.060) = 5870.8
Control group	8.00 ± 0.64	7.40 ± 1.03	7.20 ± 0.75	6.40 ± 0.49	*P *< 0.05 (0.88)	*P *< 0.05 (0.987)
Lysholm						
USG-DN group	56.00 ± 4.05	66.20 ± 5.10	74.40 ± 3.92	83.40 ± 5.78	*F* (66.72; 68.42) = 2146.62	*F* (3.143; 6.557) = 24,851.05
Control group	55.20 ± 2.16	59.60 ± 3.24	68.60 ± 5.10	80.20 ± 4.54	*P *< 0.05 (0.965)	*P *< 0.05 (0.997)
VISA-P						
USG-DN group	43.10 ± 7.08	52.30 ± 11.60	67.20 ± 9.33	88.70 ± 8.59	*F* (58.18; 61.21) = 856.12	*F* (3.22; 9.27) = 6160.55
Control group	40.70 ± 6.61	49.30 ± 8.07	58.10 ± 5.17	78.20 ± 8.34	*P *< 0.05 (0.916)	*P *< 0.05 (0.987)
KOOS pain						
USG-DN group	54.55 ± 8.50	65.40 ± 5.45	74.40 ± 4.90	87.18 ± 6.96	*F* (67.25; 69.43) = 596.67	*F* (1.87; 6.24) = 15,475.55
Control group	51.28 ± 7.6	63.20 ± 5.77	71.40 ± 4.64	79.40 ± 6.19	*P *< 0.05 (0.884)	*P *< 0.05 (0.995)
KOOS symptoms						
USG-DN group	55.60 ± 8.09	65.20 ± 7.50	73.00 ± 5.47	85.60 ± 4.27	*F* (66.87; 69.37) = 1048.74	*F* (0.95; 5.95) = 11,771.375
Control group	53.80 ± 6.76	61.40 ± 7.83	68.60 ± 5.49	81.80 ± 3.75	*P *< 0.05 (0.931)	*P *< 0.05 (0.993)
KOOS ADL						
USG-DN group	56.56 ± 7.12	68.17 ± 4.96	76.55 ± 3.25	89.29 ± 5.19	*F* (68.52; 70.43) = 676.089	*F* (4.41; 8.229) = 21,031.647
Control group	51.29 ± 7.6	63.20 ± 5.77	71.40 ± 4.64	79.40 ± 6.19	*P *< 0.05 (0.897)	*P *< 0.05 (0.996)
KOOS sports recreational activity						
USG-DN group	59.20 ± 8.90	68.40 ± 8.30	76.60 ± 3.61	86.60 ± 4.27	*F* (68.47; 70.72) = 881.642	*F* (4.143; 9.057) = 12,755.399
Control group	56.80 ± 6.76	61.40 ± 7.83	68.60 ± 5.49	81.80 ± 3.75	*P *< 0.05 (.919)	*P *< 0.05 (0.994)
KOOS QOL						
USG-DN group	54.55 ± 8.50	65.40 ± 5.45	74.40 ± 4.90	87.18 ± 6.96	*F* (67.25; 69.44) = 596.607	*F* (1.87; 6.24) = 15,475.79
Control group	51.28 ± 7.6	63.20 ± 5.77	71.40 ± 4.64	79.40 ± 6.19	*P *< 0.05 (0.884)	*P *< 0.05 (0.995)
KOOS total						
USG-DN group	56.56 ± 7.12	68.17 ± 4.96	76.55 ± 3.25	89.29 ± 5.19	*F* (68.52; 70.43) = 676.089	*F* (4.41; 8.229) = 21,031.647
Control group	51.29 ± 7.6	63.20 ± 5.77	71.40 ± 4.64	79.40 ± 6.19	*P *< 0.05 (0.897)	*P *< 0.05 (0.996)

Analysis included repeated-measures analysis of variance with baseline measurement as variate with Bonferroni corrections applied.

DF, degrees of freedom; USG-DN, ultrasound guided dry needling; SD, standard deviation; VAS, visual analog scale; VISA-P, Victorian Institute of Sports Assessment for PT, KOOS, Knee Injury and Osteoarthritis Outcome Score; ADL, function in daily living; QOL, quality of life.

^a^ Estimating Greenhouse–Geisser. Partial eta^2^ (effect size).

(Statistically significant = *P*-value ≤ 0.05). A statistically significant difference was observed.

### Functional disability

VISA-P, Lysholm, and KOOS values increased significantly (*P*-value ≤ 0.05) over time irrespective of the group. This effect was significantly stronger in USG-DN + CPT group compared with the CPT group.

### Ultrasonographic assessment of patellar tendon

**Figure 2:**
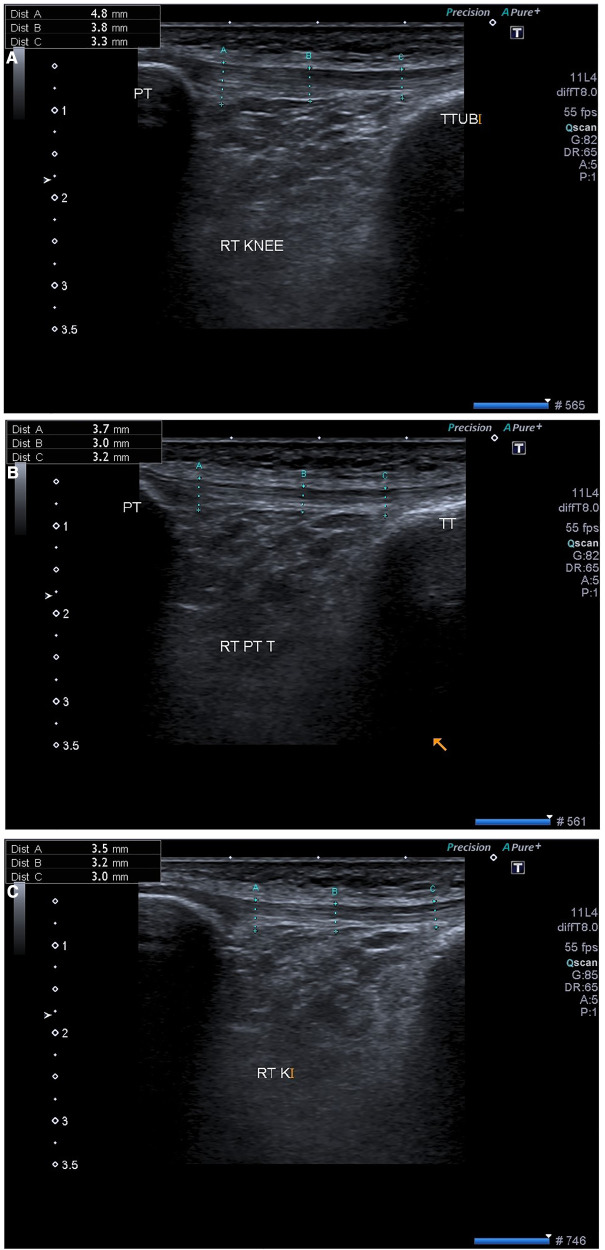
Ultrasonographic images from a 27-year-old Male with patellar tendinosis and VAS scores of 6 (pre-procedure), after (**A**) 1st week (**B**) 2nd week and (**C**) 4th week in the same patient. The post-intervention sonographic image confirms decreased tendon thickness and the hypoechoic segment resolved in the patellar tendon VAS score of 3 (post-procedure). VAS, visual analog scale.

The patellar tendon was measured in long and short axis. There was a visible heterogeneous hypoechoic on both the longitudinal and transverse images. The patient’s knee was kept in 30 flexions and the transducer of the ultrasound was moved back and forth to rule out an anisotropic effect. The normal patellar tendon appears fibrillar under sonographic assessment which becomes disrupted after inflammation. The patellar tendon was focused on long axis view and three measurements were taken:

(1) Proximal within 1 cm of patella
(2) Distal within 1 cm at insertion on tibial tuberosity
(3) Middle of patellar tendon as shown in Figure 2.

Post-therapy imaging (USG-DN + CPT) was performed with the same technique, knee position, and machine as in the pre-treatment examination. The sonographic features that were used for patellar tendinopathy diagnosis were reassessed for interval change. These included tendon thickness, presence and size of the central hypoechoic area, echogenicity, fibrillar echo pattern, and color doppler activity. It was observed a decrease in patellar tendon thickness, width, and a slightly increased echogenicity in the previously heterogeneous hypoechoic area within the patellar tendon. Furthermore, during the sonographic assessment, the inflamed patellar tendon showed disruption of the fibrillar echo pattern, decreased echogenicity in the focal region, and increased vascularity on doppler ultrasound in a few patients. There was a statistical difference found between both groups at 1 month regarding ultrasound characteristics, Table 4.

**Table 4:** Results of ultrasonographic examination.

Outcome	Grouping variable	*N*	USG-DN + CPT Group		CPT Group		USG-DN + CPT VS CPT	
			Mean ± SD	SE	Mean ± SD	SE	Mean difference (95% CI)	*P*-value
Tendon thickness, mm	Baseline	47	7.51 ± 0.23	0.03	7.65 ± 0.13	0.01	−0.14 (−0.22 to −0.06)	0.002
	Fourth week	47	0.70 ± 0.34	0.05	1.90 ± 0.32	0.04	−1.20 (−1.34 to −1.06)	0.001
Thickness of central hypoechoic area, mm	Baseline	47	4.42 ± 0.28	0.04	4.62 ± 0.30	0.04	−0.20 (−0.32 to −0.07)	0.001
	Fourth week	47	0.50 ± 0.33	0.04	1.65 ± 0.29	0.04	−1.14 (−1.27 to −1.01)	0.001
Baseline central hypoechoic area present	Baseline	47	0.80 ± 0.39	0.05	0.10 ± 0.31	0.04	0.70 (0.55–0.84)	0.003
	Fourth week	47	0.89 ± 0.31	0.04	0.31 ± 0.47	0.06	0.57 (0.41–0.73)	0.002
Baseline Colour Dopler activity (grade 0–4)	Baseline	47	2.55 ± 1.23	0.17	0.63 ± 0.94	0.13	1.91 (1.46–2.36)	0.001
	Fourth week	47	2.76 ± 1.37	0.19	2.00 ± 0.78	0.11	0.76 (0.30–1.22)	0.001

USG-DN, ultrasound guided dry needling; CPT, conventional physical therapy; SD, standard deviation; SE, standard error; CI, confidence interval; (Statistically significant = *P*-value ≤ 0.05).

## Discussion

PT is the most common knee pathology with a high prevalence of approximately 13%–20% among athletes (48–50). The most striking finding is the absence of high-quality adequate treatment that demonstrates superior outcomes than the conventional modalities or interventions. There are several options for the treatment of patellar tendinopathy with unclear efficacy (51).

The current study was designed based on previously reported interventions for the treatment of patellar tendinopathy. In this proposed study, the effects of invasive treatment have been analyzed and discussed in physical therapy for tendinopathy. In our current study, four ultrasonographic features of patellar tendinopathy were used for diagnosis and follow-up. These include tendon thickness, tendon width, echogenicity, and fibrillar echo pattern. The principal findings of the study showed a statistically significant reduction in pain intensity at 1 week post-treatment, the adjusted mean VAS was 0.4 points higher in the control group as compared to the ultrasound guided DN group, (*β* = 0.4, SD = 0.13, *P*-value ≤ 0.05); at 1 month, this difference had grown to 3.4 points (*β *= 3.4, SD = 0.94, *P*-value ≤ 0.05; Table 2). At 1 week post-treatment, the adjusted average VISA-P score was 3.0 points higher in the ultrasound guided DN group as compared to the control group (*β *= 3.0, SD = 3.53, *P*-value ≤ 0.05); at 1 month, this difference had grown to 10.5 points (*β *= 10.5, SD = 0.25, *P*-value ≤ 0.05; Table 2). At 1 week post-treatment, the adjusted mean Lysholm score was 6.6 points higher in the ultrasound guided DN group as compared to the control group (*β *= 6.6, SD = 1.86, *P*-value ≤ 0.05); at 1 month, this difference had grown to 7.2 points (*β *= 7.2, SD = 0.25, *P*-value ≤ 0.05; Table 2). At 1 week post-treatment, the adjusted mean KOOS total score was 5.51 points higher in the ultrasound guided DN group as compared to the control group (*β *= 5.51, SD = 0.81, *P*-value ≤ 0.05); at 1 month, this difference had grown to 9.9 points (*β *= 9.89, SD = 1, *P*-value ≤ 0.05; Table 2).

It has been analyzed that ultrasound guided DN is an effective intervention modality for the treatment of patellar tendinopathy. This finding was consistent with a previous case study describing that ultrasound guided DN showed improvement in pain and function in patellar tendinopathy (22). It has been reported in a systematic review, that revealed DN as an important adjunct treatment in the conservative management of tendinopathy (52), whereas one previous study contradicts the current results and states that DN or PNE combined with EE has not proved to be more effective than a program of only EE in improving disability and pain in patients with patellar tendinopathy in the short term of 10 weeks and medium term of 22 weeks. All clinical improvements were not associated with structural tendon changes (53).

Several conventional interventions have been used for tendinopathy and are found to be non-reflective to address any destruction to the healthy tissues. Several conventional interventions such as eccentric muscle strengthening, iontophoresis, ultrasound, phonophoresis, low-level laser treatment, and extracorporeal shockwave therapy have been advocated for the treatment of patellar tendinopathy (54). The effectiveness of different conventional interventions appears to be unproven for the treatment of patellar tendinopathy. The relatively new treatment modality-ultrasound guided DN procedure is promising to be involved in visualization under sonography to uncover the pathological tissues in patellar tendinopathy. For example, DN coupled with autologous blood injection showed promising results under ultrasound guidance (VISA score: mean pre-procedure score = 39.8 (range 8–72) *v* mean post-procedure score = 74.3 (range 29–100), *P *< 0.001; mean follow up 14.8 months (range 6–22 months) (1). In another study, it has been reported that ultrasonography guided treatments facilitate the development of percutaneous procedures of regenerative healing in tendinopathy (55). The efficacy of DN under the guidance of sonography has been evaluated in several reported studies and found to be statistically significant for the treatment of tendinopathy, rotator cuff, and tendons around the greater trochanter (54). Hence, it is demonstrated that this technique can be adopted and utilized as a first-line therapy or as a secondary treatment for the treatment of tendinopathy. In addition to this, ultrasound guided DN was found promising to recognize the degenerative tissue within the tendon substance.

The tendinous tissue takes a long time for repair and regeneration and therefore, 1-month observation in the current study was too short to demonstrate ultrasound changes. However, in a previous study, the use of corticosteroid injection has shown significant reduction in tendon thickness after only 1 month (56). So, US is considered an important imaging modality to include in clinical trials and is widely used to clinically diagnose at an initial stage (57, 58). The US image has shown change over time without a strong relation to symptoms (59–61). The inclusion of US in clinical trials is very important to get knowledge about variation in imaging over time and its relation to symptoms.

Although our study design does not permit us to comment on the mechanism of action of this technique, it is recognized that DN mechanically disturbs the scar tissue through repeated fenestration of the tendon, which leads to localized bleeding (62, 63). This bleeding into the newly fenestrated tendon promotes healing by stimulating the growth factors (64). This healing cycle is mediated by different growth factors such as transforming growth factor-*β* and basic fibroblastic growth factor and both are found in the blood (1, 65). The healing cycle induces remodeling within the tendon structure and regains the majority of its mechanical properties (66). The healing of the tendon by growth factors promotes cellular proliferation and increases matrix production (67). These changes led the author to make an opinion that this cellular proliferation and matrix production are responsible for the increased echogenic signal within the treated tendon and cause pain reduction in patients, therefore mechanical properties of the tendons are regained without different interventions over a longer duration.

The sonographic features of patellar tendinopathy are well-known (68). Abnormal hypoechogenicity of the patellar tendon with inconsistent blood flow on color or power Doppler imaging confirms the diagnosis of tendinosis (64). The posterior aspect of the proximal patellar tendon involvement is common, with hyperechoic and shadowing calcifications alongside cortical irregularity of the patella may be observed. In our study, with reference to the sonographic findings of patellar tendinopathy, there was a statistically significant correlation between well-defined margins of patellar tendon abnormality and a positive clinical response to ultrasound guided tendon DN at 4 weeks, demonstrated by an improved pain and function scores. The rest of the sonographic features related to color or power Doppler imaging did not represent a statistical correlation with a clinical outcome.

There are certain limitations in the study. Although VISA-P, Lysholm, and KOOS scales were used to determine the objective measures of clinical outcomes in patients treated with USG-DN, we lacked any other objective measurement of tendon healing. The sonographic values are descriptive rather than subjective. It would be hard to justify tendon biopsy to find histological evidence of tendon healing. By keeping this in view, ultrasound was selected as a method to observe the “healing response” and determined the ultrasonographic features of the tendons treated by this innovative technique. Further randomized controlled trials of USG-DN are required to meet the challenges and optimization of the proposed study. Second, this study only revealed a 1-month follow-up. Therefore, we are unable to confirm the long-term effectiveness of USG-DN in reducing symptoms of tendinopathy. Further research is required to determine the long-term effects of ultrasound guided DN, compared with other techniques. In addition, future high-quality studies are recommended to investigate the mechanical properties of tendons after DN therapy in individuals with tendinopathies, which is one of the major concerns in daily clinical practice. Lastly, the study included only a young population with a range of 18–45 years, therefore the results cannot be generalized to all patients of patellar tendinopathy. It is also suggested to perform the USG-DN technique in other tendons and measure the long-term effects.

## Clinical and research implications

The current study offers an idea of how to manage better, the treatment of tendinopathy using ultrasound guided DN technique. Knowing a little more about the behavior of the pain in relation to the affected area, the problems of our patients can be addressed with more efficient treatments.

A substantial contribution is required in this line of work in which the sample of participants would be expanded. Several sessions for the tendon treatment and a more drawn-out term follow-up both are under discussion.

## Conclusion

In conclusion, the study showed that 1 month of ultrasound guided DN was clinically effective in reducing pain in patients with patellar tendinopathy. Ultrasound guided DN technique resulted in statistically significant improvement in knee function and ultrasonographic features of patellar tendinopathy among patients. The improvement had a low to moderate effect and less clinically significant improvement in the control group receiving CPT. Patients can improve their function and reduce their disability with this intervention.

## Data Availability

The raw data supporting the conclusions of this article will be made available by the authors, without undue reservation.

## Supplementary materials

CONSORT guidelines. All methods were performed following the relevant guidelines and regulations. (Supplementary Materials).
